# Statistical analysis plan for a parallel group randomized clinical trial comparing schema therapy versus treatment as usual for outpatients with difficult-to-treat depression (DEPRE-ST)

**DOI:** 10.1186/s13063-025-09012-4

**Published:** 2025-09-01

**Authors:** Ida-Marie T. P. Arendt, Matthias Gondan, Sophie Juul, Lene Halling Hastrup, Carsten Hjorthøj, Stine B. Moeller

**Affiliations:** 1 Department of Psychology, University of Southern Denmark, Campusvej 55, 5230 Odense, Denmark; 2 Department of Interdisciplinary Trauma Treatment, Mental Health Services in the Region of Southern Denmark, Østre Hougvej 55, 5500 Middelfart, Denmark; 3 Department of Psychology, Universität Innsbruck, Innrain 52, 6020 Innsbruck, Austria; 4 Centre for Clinical Intervention Research, Copenhagen Trial Unit, Copenhagen University Hospital, Blegdamsvej 9, 2100Copenhagen Ø Rigshospitalet, Denmark; 5 Research Unit of Stolpegaard Psychotherapy Centre, Mental Health Services, Capital Region of Denmark, Stolpegaardsvej 20, 2820 Gentofte, Denmark; 6 Department of Psychology, University of Copenhagen, Øster Farimagsgade 2a, 1353 Copenhagen, Denmark; 7 Psychiatric Research Unit, Psychiatry in Region Zealand, Faelledvej 6, 4200 Slagelse, Denmark; 8 Danish Centre for Health Economics (DaCHE), University of Southern Denmark, Campusvej 55, 5230 Odense M, Denmark; 9 Copenhagen Research Center for Mental Health – CORE, Mental Health Services in the Capital Region, Copenhagen, Denmark; 10 Department of Public Health, Section of Epidemiology, University of Copenhagen, Øster Farimagsgade 5, 1353 Copenhagen K, Denmark

**Keywords:** Schema therapy, Difficult-to-treat depression, Treatment-resistant depression, Treatment refractory depression, Chronic depression, Depressive personality disorder, Persistent depressive disorder, Randomized controlled trial, Psychotherapy, Childhood trauma

## Abstract

**Background:**

“Difficult-to-treat” depression — here operationalized as either chronic or treatment-resistant depression — encompasses about one third of all patients with depression. Despite its considerable size, the patient group is understudied, and evidence-based psychotherapeutic treatment options are currently limited. The DEPRE-ST trial therefore seeks to investigate beneficial and harmful effects of schema therapy for difficult-to-treat depression.

**Methods:**

This is a randomized multicenter parallel-group superiority clinical trial. A total of 129 patients will be randomized to receive either 30 sessions of individual schema therapy or psychotherapy according to Treatment As Usual, along with psychopharmacological treatment. Participants are assessed before randomization and at 6, 12, and 24 months after randomization. The primary outcome is depression symptoms (6-item Hamilton Rating Scale for Depression), assessed at 12 months. Secondary outcomes include health-related quality of life (European Quality of Life 5 Dimensions 5 Level Version), functional impairment (Work and Social Adjustment Scale), psychological wellbeing (WHO-5 Well-being Index), and negative effects of treatment (Negative Effects Questionnaire), alongside a range of patient-relevant exploratory outcomes. Blinding of treatment allocation is ensured for primary outcome assessors, statisticians, and the data safety and monitoring committee. The primary outcome will be analyzed with multilevel linear regression, with conservative multiple imputation for missing data, and presented as the covariate-adjusted difference between treatments’ change scores with its 95% confidence intervals. Further, a health economic analysis will be performed.

**Discussion:**

This statistical analysis plan was developed and submitted before unblinding of data to ensure transparency and diminish bias in selection, analysis, and reporting of results.

**Trial registration:**

ClinicalTrials.gov NCT05833087. Registered on 15th April 2023. https://clinicaltrials.gov/study/NCT05833087?term=depre-st&checkSpell=false&rank=1

**Supplementary Information:**

The online version contains supplementary material available at 10.1186/s13063-025-09012-4.

## Introduction

### Background and rationale

While major depression in general is seen as treatable with many forms of psychotherapy [1], some patients require more specialized and focused forms of psychotherapy treatment [2, 3]. Up to 35% of patients suffer from either chronic (> 2 years duration) or treatment-resistant depression (often defined as persistent depression after sufficient treatment attempts with at least two different antidepressants), which together can be labelled “difficult-to-treat depression” (DTD) [4]. Despite both high prevalence and illness burden, DTD is underprioritized in psychotherapy research, with room for improvement both in the understanding of the disorder and in efficient treatment options [5, 6].

Patients with DTD seem to differ qualitatively from patients with non-DTD in several aspects [7]: First, they exhibit much higher frequencies of childhood adversity [8–11]. Second, problematic personality traits or comorbid personality disorders as well as dysfunctional interpersonal behavior or cognitive styles are more common [10, 12]. As all of these factors can both exacerbate depression symptoms, maintain or prolong depressive periods, and be risk factors for depression relapse [8–14], they should be addressed in psychotherapeutic treatment for DTD in order to ensure a strong and long-lasting treatment effect.

Schema therapy (ST) was derived from cognitive behavior therapy with elements from psychodynamic, emotion focused, and Gestalt therapy, exactly with the purpose of treating more complex cases with deeply rooted psychological issues. Using a distinct experiential focus [15, 16], ST works directly with childhood adversity and trauma, while it trans-diagnostically addresses problematic personality traits underlying the presenting symptoms and disorders. While some smaller studies on schema therapy for chronic depression have shown promising results [17, 18], and two recent randomized controlled trials have investigated the effects of ST compared to psychodynamic therapy for depression with comorbid borderline personality disorder [19] and the effects of ST for depression more broadly [20], ST for DTD has to our knowledge not yet been studied in a randomized controlled trial.

The current trial aims to contribute to the evidence base for ST in the treatment of DTD. It investigates the beneficial and harmful effects of up to 30 individual sessions of ST compared to treatment as usual (TAU) for depression in a secondary psychiatric out-patient setting.

## Methods

### Trial design

DEPRE-ST is a multi-center, two-arm, parallel group, assessor-blinded, randomized controlled superiority study, comparing the effect of ST vs TAU on the primary outcome of depression symptoms (HAMD-6) at 12 months after randomization. Patients are allocated 1:1 to either 30 sessions of individual ST or TAU for major depression. TAU can be delivered both as individual and group therapy and may be psychodynamic or cognitive behavior therapy.

### Trial interventions

*Schema therapy* will consist of 30 weekly 45–60-min individual sessions of ST during a period of 10 to 12 months (allowing for sick days and holidays). The therapy is delivered according to a manual developed for this study (see study protocol [21]). *Treatment As Usual* corresponds to the psychotherapeutic treatment that is routinely offered at the particular treatment site. It can consist of Cognitive Behavioral Therapy or psychodynamic therapy, either individually (6–16 sessions of 45–60 min) or in groups (13–16 sessions of 90–120 min).

Since the study protocol was submitted [21], two more sites have been added to the trial: a district psychiatric out-patient center in the Danish town Svendborg, and a psychiatric out-patient clinic specializing in treatment of mood disorders in the Danish capital Copenhagen.

In both treatment arms, participants will additionally be offered psychopharmacological treatment (as deemed appropriate for the individual patient), meetings with next-of kin, etc., as these are parts of the treatment in the Danish secondary mental health system. See the study protocol [21] for more details on treatment, both specific and general, for the two treatment arms.

### Eligibility

*Participant eligibility criteria* are as follows (identical to those stated in the study protocol [21]):

- Outpatient
- Aged 18 or above
- Referred to treatment for depression in a psychiatric clinic, or already in treatment at the clinic and eligible for a second treatment package at the time of inclusion. For an explanation of treatment packages, see Item 11a of the study protocol [21].
- Meeting the diagnosis of chronic or treatment-resistant depression, that is: Clinical major depressive disorder (MDD) as assessed by the M.I.N.I.−5 diagnostic interview [22]; MDD duration of at least two years *or* persistent MDD after two or more trials of antidepressants from different classes, in an adequate dosage and time period (≥ 4 weeks), *or* moderate treatment resistance as measured on the Maudsley Staging Model (MSM) [23], score above 6.
- Minimum score of 9 points on the Hamilton Rating Scale for Depression 6 (HAMD-6), corresponding to moderate to severe MDD.

*Exclusion criteria* are as follows:

Concurrent with the criteria for treatment packages for depression in Danish secondary mental health settings:

- Alcohol or substance abuse
- Bipolar disorder
- Psychotic disorders or current psychotic symptoms of a character that precludes a depression treatment package
- Acute suicidal risk
- Mental disability (estimated IQ < 70)

Further study-specific exclusion criteria:

- Non-Danish speaker, to the extent that the person cannot read and respond to the trial questionnaires in Danish
- Have received treatment with ST in the past 5 years
- Pregnancy known at the time of inclusion, since childbirth and infant care could lead to difficulties in upkeeping continuous treatment appointments.

#### Screening of eligible participants

**Fig. 1:**
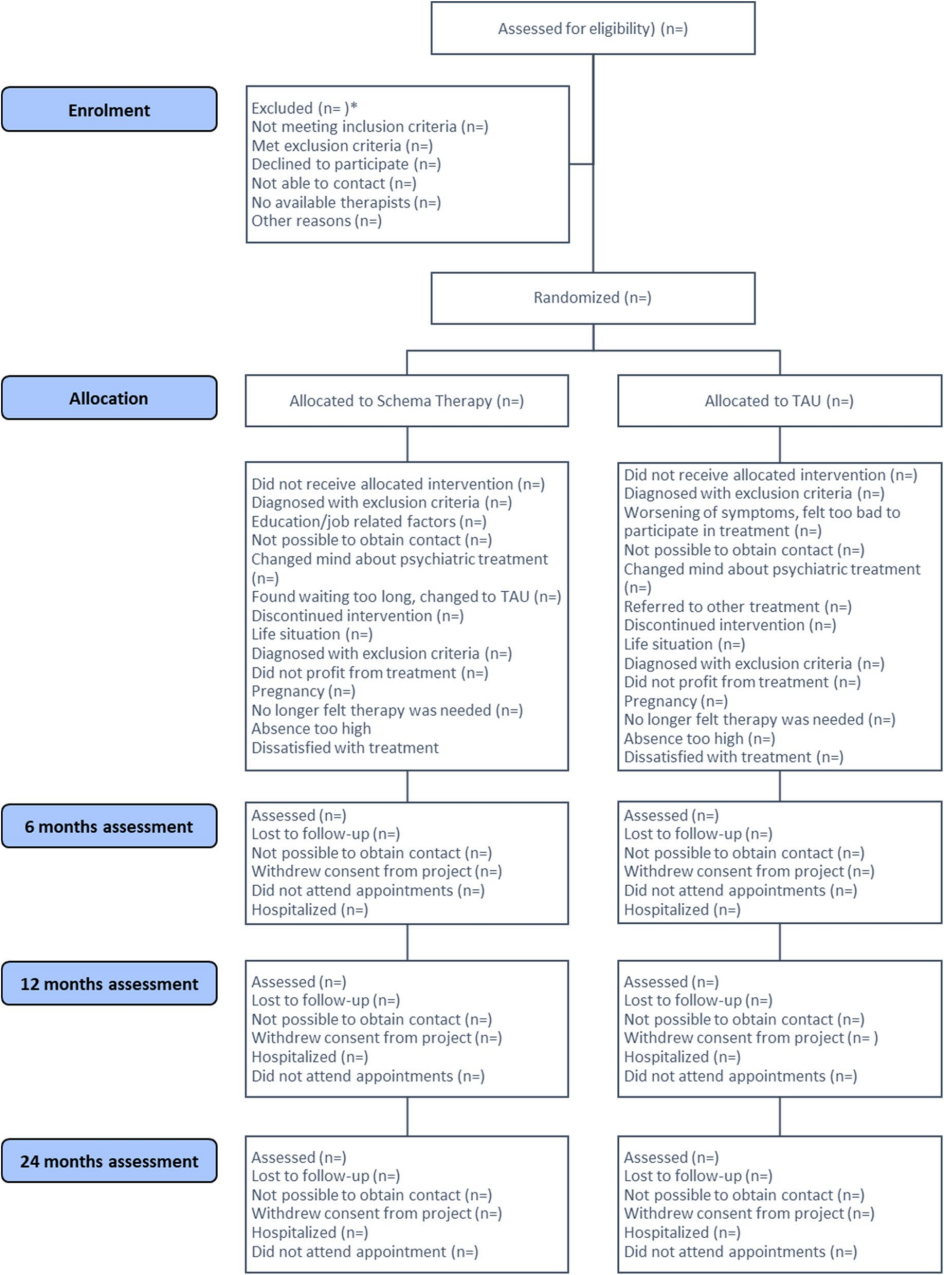
Consolidated Standards of Reporting Trials (CONSORT) flow chart

Eligible patients are identified at the participating sites, either by researchers present at multidisciplinary case conferences or by a treatment provider at the site, whereafter a short phone screening for eligibility takes place. If the participant is deemed initially eligible, a more thorough eligibility screening is made in person or in an online meeting, after which the patient can be included and randomized. We will register the overall number of potentially eligible patients that we have screened in conferences or through referral from treatment providers, the number of patients not deemed eligible for participation, and overall reasons for not including patients (e.g., did not fulfill the inclusion criteria). All information is entered in a CONSORT flow diagram (see Fig. 1). We will not be able to register the total number of potentially eligible patients for the study as we are not able to be present at all case conferences throughout the study, meaning that this number would be unreliable.

### Patient characteristics

The following data will be collected at baseline: age, sex, country of origin (Danish/non-Danish, Western/non-Western), civil status, living alone or with partner/children/others, number of children, level of education, current job situation (including sick leave), yearly income of patient and of household, current psychosocial stressors (Yes/No), number of previous depressions, age at the time of the first depression, former psychiatric treatment (duration, format), depression and other diagnoses from electronic patient journal, comorbid diagnoses according to the M.I.N.I diagnostic interview, severity of current depression measured on the HAMD-6 (grouped by severity in “Moderate depression” or “Severe depression”), current and former intake of psychotropic medicine in current depressive episode, former treatment with psychotherapy in current depressive episode, other treatments in current depressive episode, childhood adversity, expectancies for changes in depression symptoms, and the baseline measurements of the primary, secondary, and exploratory outcomes. Baseline characteristics will be presented in a table as seen in Additional file 2.

### Adherence

Criteria for protocol adherence are defined at the level of both the therapists and the patients.

#### Therapist adherence

All ST therapists’ competency and adherence to the treatment protocol is ensured in several ways: ST training consists of formalized, practice-based live and online training, tailored to the current level of ST knowledge for the individual therapist. Initially, one video recording is submitted by each therapist with a ST session with a current patient at the treatment site (who is not participating in the study). Therapists performing below the required level of competency will be asked to resubmit a video recording of a session with ST until satisfactory standards are met.

All schema therapists receive 1.5 h of group supervision monthly throughout the treatment phase of the study. One-third of all sessions with schema therapy are videotaped. The therapist adherence to the schema therapy manual and therapists’ competency in schema therapy is measured by rating two randomly selected recordings from each patient with the Schema Therapy Rating Scale [24]. Adherence will be reported as the number of schema therapy elements used in the session (number of endorsed items from items number 6–14), while competency will be reported as the average grade given for all endorsed items.

#### Patient adherence

Major protocol deviations are defined as:

- Premature drop-out of treatment without prior agreement with the therapist. However, discontinuation of treatment is not noted as dropout in the case of explicit agreement between therapist and patient due to either persistent improvement of the patient’s condition, lack of effect or adverse effects, low patient motivation, treatment adherence or attendance, or referral to other treatment due to exclusion criteria that were not known at the time of inclusion (for details, please consult the study protocol [21]).
- Cross-over, that is, the patient is randomized to one study arm but received the treatment from the other arm.
- Contamination, that is, patients given treatment or elements of treatment from the other treatment arm in addition to the treatment they were randomized to. This does not include elements of Cognitive Behavior Therapy (CBT) given as part of a schema therapy treatment, as CBT elements (such as cognitive restructuring) can be part of ST and are included in the ST manual.
- Non-permitted additional treatment, for example, patients given additional treatment at the treatment site than the treatment they have been randomized to receive (e.g., CBT for insomnia).
- Other deviations that might arise during the course of the trial will be reported if deemed that they can influence the effects of treatment (exceptions to these are noted in the study protocol [21].

The electronic patient journals are accessed in order to record the above information. The absolute number and percentage of treatment drop-out, crossover, contamination, and other deviations, and the average length of treatment (completed sessions) will be reported in the main article.

Participants may drop out from the intervention but still participate in data collection; they may continue in the intervention but no longer wish to participate in data collection; or they may be lost to follow-up in the case we are not able to obtain contact with them. However, they are not able to withdraw consent to the analysis of data already collected. Level, timing of and reasons for withdrawal, and lost to follow-up will be presented in the CONSORT flow diagram (see Fig. 1). In the case we cannot get in touch with a participant, we will get data from the national Danish registries to see whether the participant may have moved abroad or died.

##### Randomization and stratification

Randomization is done directly after completion of the baseline assessment in the data management system REDCap (Research Electronic Data Capture), a secure web application which is used in the trial for building and managing measurement tools. Outcome assessors (for the observer-rated HAMD-6), data managers, statisticians, the data safety monitoring committee, and researchers writing up overall conclusions for the outcome article are all blinded to treatment allocation. For details about the timing about randomization and sequence generation as well as information on blinding, see the study protocol and its Additional file 4 [21].

The randomization was initially stratified by depression severity (moderate or severe; HAMD-6) and childhood adversity (Childhood Trauma Questionnaire (CTQ-SF), dichotomized as a CTQ score of < or ≥ 49) at baseline. However, after including 14 participants, the trial team realized that stratification for treatment site was lacking, where after this stratification has been applied for the remainder of the included participants, in lieu of stratifying for depression severity and childhood adversity.

##### Sample size

The sample size was calculated based on the primary outcome, HAMD-6, with a minimally clinically relevant difference of 2.0 points between the treatment groups and a standard deviation of 3.5. For further details of the sample size calculation, see the study protocol [21]. The total sample size will be 129 participants, giving 80% statistical power when accounting for approximately 20% dropout as well as clustering for about 5 patients per therapist, assuming an intra-cluster correlation of 0.01. Table 1 shows that this number of patients also yields adequate power in the secondary outcomes, but not for the exploratory outcomes [25–34].

**Table 1:** Power estimates for secondary and exploratory outcomes

**Outcome**	**MCID**	**SD**	$\boldsymbol{\alpha }$	**Power**	**Reference**
*Secondary*					
Level of functioning (WSAS)	8.0	9.59	5%	98%	[27]
Psychological well-being (WHO-5)	10.0	7.9	5%	100%	[26]
Health-related quality of life (ED-5D-5L)	0.071	0.102	5%	93%	[28]
Negative effects of treatment (NEQ)	2.0	3.5	5%	89%	[29]
*Exploratory*					
Changes in patient-elected problems (PSYCHLOPS)	2.33*	4.66	5%	70%	[30]
Perseverative Thinking (PTQ)	5.6*	11.2	5%	70%	[31]
Anger reactions (DAR-7)	6.8*	13.6	5%	70%	[32]
Anxiety (SCL-10, anxiety subscale)	0.31*	0.71	5%	70%	[33]
Anger processing (MAP)**	-	-	-	-	-
Personal recovery (Brief INSPIRE-O)	8	20.7	5%	48%	[34]

*MCID,* minimal clinically relevant difference *SD,* standard deviation, α: two-tailed significance level. For simplicity, the above power estimates do not account for clustering or study dropout.

*Where the MCID was not available, it was estimated as SD/2

**It was not possible to obtain data for the MAP

## Outcomes

For details on the outcomes, please see the study protocol [21]. Outcomes are assessed at 6, 12, and 24 months after randomization (with a few exceptions, see Table 2). The primary time point of interest is 12 months after randomization for all outcomes with a few exceptions (for details, see Table 2). It is the goal to have a “visit window” of no more than 4 weeks around the exact planned time point for assessment. The average timing (days) between randomization and outcome assessments will be reported.

**Table 2:** Timing of interventions and assessments

	Enrollment	Post-allocation				Planned
	T0	Start treatment	Month 6	Month 12	Month 24	Month 36
Eligibility screening	X					
Informed consent	X					
Randomization	X					
Schema therapy						
Treatment as usual						
Baseline data	X					
PSYCHLOPS pre	X					
PSYCHLOPS post			TAU	ST		
CTQ	X					
DCES-P	X					
NEQ			X	X	X	X
EQ-5D-5L	X		X	X	X	X
PTQ	X		X	X	X	X
WSAS	X		X	X	X	X
MAP-9	X		X	X	X	X
DAR	X		X	X	X	X
WHO5	X		X	X	X	X
INSPIRE-O Brief	X		X	X	X	X
SCL-10	X		X	X	X	X
SMI 1.1 (part)	TAU		X	TAU	X	X
YSQ S.3 (part)	TAU		X	TAU	X	X
SMI 1.1	ST			ST		
YSQ S.3	ST			ST		

*CTQ* Childhood Trauma Questionnaire, *DAR* Dimensions of Anger Reactions Scale, *DCES-P* Depression Change Expectancy Scale—Pessimistic scale, *EQ-5D-5L *European Quality of Life 5 Dimensions 5 Level Version, *MAP-9* Metacognitive Anger Processing Scale (9 items), *NEQ* Negative Effects Questionnaire, *PSYCHLOPS* post Psychological outcome profiles post-therapy, *PSYCHLOPS* pre Psychological outcome profiles pre-therapy, *PTQ* Perseverative Thinking Questionnaire, *SCL-10* Symptom Checklist (anxiety subscale; 5 items), *SMI* 1.1 Schema Mode Inventory version 1.1 (full version), *SMI* 1.1 part Schema Mode Inventory version 1.1 (Healthy Adult, Vulnerable Child, Punitive and Demanding Parent-modes only), *ST* schema therapy, *TAU* treatment as usual, *WHO-5* WHO-5 Well-Being Index, *WSAS* Work and Social Adjustment Scale, *YSQ S.3* Young Schema Questionnaire S3 (full version), *YSQ S.3* part Young Schema Questionnaire S3 (Emotional Deprivation, Abandonment, Mistrust/Abuse, Social Isolation, and Defectiveness schemas only).

### Primary outcome

The primary outcome is the severity of depression, assessed with the 6-item Hamilton Rating Scale for Depression (HAMD-6) [35], observer-rated, 12 months after randomization. The HAMD-6 will also be assessed at baseline (for covariate adjustment), and at 6 and 24 months after randomization (as a secondary outcome).

### Secondary outcomes

In recent years, there has been increasing focus on the voice of patients and their needs [36]. Especially in the case of severe or chronic illnesses, full recovery from symptoms might not be feasible, but treatments may provide other beneficial effects that are equally or more important for patients. For this reason, the current study has chosen a wide array of secondary and exploratory outcomes, as seen below, in the effort to encompass alternative ways of measuring treatment success. Notably, the Psychological Outcome Profiles (PSYCHLOPS) [30]) lets the patient choose idiosyncratic problems that might be targeted by treatment.

- Depression symptoms on the HAMD-6 at 6 and 24 months after randomization
- Quality of life, assessed with the European Quality of Life 5 Dimensions 5 Level Version (EQ-5D-5L) (primary time point of interest at 24 months after randomization)
- Level of functioning, assessed with the Work and Social Adjustment Scale (WSAS)
- Psychological well-being, assessed with the WHO-5 Well-Being Index (WHO-5)
- Negative effects of treatment, assessed with the Negative Effects Questionnaire (NEQ)

### Exploratory outcomes

Exploratory outcomes were defined as such on the basis of inadequate power (< 80%) [25] (see Table 1). Similar to the secondary outcomes mentioned above, the results on the exploratory outcomes will be interpreted in a non-confirmatory, hypothesis-testing sense.

- Personal recovery assessed with the Brief INSPIRE-O
- Changes in patient-elected problems assessed with the Psychological Outcome Profiles (PSYCHLOPS)
- Reactions to anger assessed with the Dimensions of Anger Reactions—Revised (DAR)
- Anger processing assessed with the Metacognitive Anger Processing scale, Short Version (MAP-9)
- Repetitive negative thinking assessed with the Perseverative Thinking Questionnaire (PTQ)
- Anxiety symptoms assessed with the anxiety subscale of the Symptom Checklist-10 (SCL-10)
- Response and remission of depressive symptoms (as defined in the study protocol [21])

Raw outcome scores will be reported, and change from baseline will be calculated where baseline measurements are available. Follow-up measurements at 36 months after randomization are planned conditional on additional funding. Outcomes from the 24- and 36-month time points, including the health economic evaluation based on the EQ-5D-5L, will be reported in separate publications.

### Safety

The proportion of participants with one or more serious adverse events will be reported for each treatment group. A serious adverse event will be defined according to the International Conference on Harmonisation of technical requirements for registration of pharmaceuticals for human use—Good Clinical Practice (ICH-GCP): any untoward medical occurrence that resulted in death, was life-threatening, required hospitalization or prolonging of existing hospitalization, and resulted in persistent or significant disability or jeopardized the participant [37]. We will collect data from the national Danish registries regarding the possible death of participants. The results of the NEQ (see above under “Secondary outcomes”) will also be analyzed to show the participants’ subjectively experienced negative consequences of treatment.

## Statistical analysis

All analyses will be done by data analysists who are blinded to treatment condition of the two groups, that is, treatment arms will be labelled “Treatment A” and “Treatment B.” Two different abstracts for the outcome article with opposing results of the two treatments and resulting conclusions will be written before unbreaking the blind, as described in the study protocol [21].

### Interim analyses

Interim analyses are planned to take place when 50% of the 6-month follow-up data have been collected. An external data safety monitoring committee (DSMC) with no allegiance to the trialists will perform the interim analyses and decide whether to stop or continue the trial based on convincing benefit or harm of the interventions. The DSMC will receive blinded data but can be unblinded upon request. Focus will be on data from the 6-month follow-up regarding serious adverse events, significant differences between groups on the primary outcome, and serious deterioration on the primary outcome.

The following statistical analyses are proposed, but the DSMC can perform other analyses and require more data if deemed necessary:

- Inferiority: A *t*-test for independent groups will be used to test for differences between study arms. If this test is significant at the one-tailed 2.5% and indicates that ST is inferior, the DSMC will reassess the costs and benefits of the trial and consider early stopping.
- Serious adverse events: A chi-square test will be used to test for differences between groups in the number of participants with one or more serious adverse events (emergency room visits for somatic or psychiatric reasons, documented suicide attempts or self-harm). If this test is significant at the one-tailed 2.5% and indicates a substantial increase in adverse events under schema therapy, the DSMC will reassess the costs and benefits of the trial and consider early stopping. In case of low event counts, Barnard’s test will be used to avoid poor asymptotic behavior of the chi-square test.
- Serious deterioration: A chi-square test will be used to test for differences between groups in the number of participants with an increase of 2 or more points on the primary outcome (HAMD-6). If this test is significant at the one-tailed 2.5% and indicates serious deterioration under schema therapy, the DSMC will reassess the costs and benefits of the trial and consider early stopping. In case of low event counts, Barnard’s test will be used to avoid poor asymptotic behavior of the chi-square test.

All interim analyses will be performed on the available data without imputation of missing data. An adjustment of the significance level is not needed, since a stop for efficacy is not planned.

### Primary analysis

The primary analysis will be based on the intention-to-treat principle (i.e., including all randomized patients who will be analyzed according to their initial randomization, regardless of the actual treatment received), using conservative, multiple imputation of missing outcomes by jump to reference [38]. The effect of the treatment will be presented as the covariate-adjusted difference between the average change scores in the two arms, with its two-tailed 95% confidence interval. In other words, superiority of ST will be claimed if the change in ST is significantly better than the change in TAU, at a two-tailed significance level of 5%. Analyses will be performed when all 12-month assessments have been completed.

The HAMD-6 score is assumed to be an interval-scaled variable, with normally distributed residuals. The therapies will be compared using a multilevel linear regression with therapy (ST vs. TAU) as the main effect, adjusted for treatment site (categorical, 6 levels) and the HAMD-6 score at baseline as covariates, and therapist as a random intercept. Efficacy of ST vs. TAU will be expressed by the covariate-adjusted difference between the change scores of the experimental versus the control intervention, along with its two-tailed 95% confidence interval. Superiority of ST will be claimed if this confidence interval is above zero. After adjustment for baseline severity, this estimate is numerically identical to the comparison of the raw scores between the treatment arms at end-of-therapy [39]. Secondary outcomes will be analyzed in the same way, with the same 95% confidence intervals. If the respective endpoint is not available in the baseline assessment by design (e.g., NEQ), the baseline HAMD-6 will be used as a baseline covariate, and effect estimates will be based on raw scores instead of change scores.

Missing outcomes will be multiply imputed by a ‘jump to the reference treatment,’ that is, we assume that patients with missing data in ST score similar to patients in TAU. This analysis method is implemented in the R package RefBasedMI [40, 41]. Box 1 (Additional file 3) shows the R code for the analysis using RefBasedMI’s own example data. Secondary outcomes will be analyzed in the same manner.

### Sensitivity analyses

To assess the impact of the imputation procedure, we will run a number of sensitivity analyses for the primary outcome (see Boxes 2 and 3, Additional file 3): A first sensitivity analysis will be based under the usual assumption of missingness-at-random. This supersedes the initially planned regression imputation with dropout status and secondary outcomes in the imputation model [21]. The reason for this change of strategy is that primary and secondary outcomes are collected at the same session. Therefore, practically speaking, missing data in secondary outcomes co-occur with gaps in the primary outcome, so imputation by secondary outcomes will not help much—it will mostly mirror the results of the analysis with available cases [42].

A second sensitivity analysis will be carried out for the subset of per-protocol participants with available outcome data, without imputation of missing outcomes. Further sensitivity analyses will be made with best-best and worst-worst case imputation of missing outcomes, and without adjustment for covariates [39].

To rule out bias due to ceiling or floor effects in the outcome, a final sensitivity analysis will be carried out using negative binomial regression, with HAMD-6 treated as count data (i.e., a score of 10 points will be counted as ten “events”) [43]. Results of this analysis will be presented as rate ratios for therapy type, adjusted for baseline severity and treatment site.

### Subgroup analyses

Subgroup analyses are performed with the purpose of assessing homogeneity of the treatment effects and further to reveal potentially harmful effects in specific subpopulations of patients. Subgroup effects will be investigated by adding treatment-by-covariate interactions to the primary analysis model and by illustrating the subgroup-specific therapy effects in forest plots (see Box 4, Additional file 3, for example code).

Patients with childhood adversity and corresponding early maladaptive schema may benefit more from ST than patients with less severe adverse experiences in childhood [44]. This will be analyzed by adding the interaction of therapy x adversity (CTQ, dichotomized as present/not present of moderate to severe childhood adversity on one or more of the five domains: physical, emotional or sexual abuse, emotional or psychical neglect, with cutoffs as suggested by Bernstein et al. [45]) to the statistical model of the primary analysis, and by estimating stratum-specific therapy effects in participants with and without adversity experiences.

Further, we will explore therapy x site interactions, as recommended in the International Conference on Harmonisation E9 guideline [46]. Additional subgroup analyses will be made for depression symptoms at baseline, sex (male, female), and psychiatric comorbidities as measured in the M.I.N.I. interview (present; not present). It is understood that such subgroup analyses are exploratory, subject to lack of statistical power and, at the same time, multiple testing issues. Any observed subgroup effect will be interpreted in the light of its psychological plausibility, weighed against statistical artifacts such as regression to the mean (e.g., treatment x severity interactions). Observed effects in the primary outcome will be compared to the corresponding results in the relevant secondary outcomes.

### Health economic analysis

The health economic analysis investigates the health-related quality of life as measured on the EQ-5D-5L and has its primary timepoint at 24 months after randomization. The EQ-5D-5L instrument includes five dimensions (mobility, self-care, usual activities, pain/discomfort, and anxiety/depression), each with five levels of severity. EQ-5D-5L responses will be converted into Quality-Adjusted Life Years (QALYs) using Danish utility weights [47].

Cost data will include direct health care costs and lost productivity costs. Direct health care costs will include costs of primary and secondary health care services and prescription medication. Unit costs of General Practitioner services and other medical specialists will be based on the prevailing National Health Insurance fee schedules [48]. Direct costs, including in-patient and out-patient health care costs, will be calculated using Diagnostic Related Groups (DRG) weights and specific out-patient tariffs. These cost estimates are all based on data from the Danish Ministry of Health and reflect the average cost of treating patients with similar conditions in a Danish hospital [49, 50]. The participants’ use of prescription medication and information on retail price on each transaction will be derived from the Register of Medical Product Statistics [51]. Lost productivity costs defined as difference in earned income between the groups and data on transfer payments will be derived from The Danish National Labour Market Authority’s DREAM database [52]. The incremental cost-effectiveness ratio (ICER) will be estimating the difference between intervention and control group in QALYs/costs [53]. Non-parametric bootstrapping will be used to estimate confidence intervals for the ICER. Results will be presented in the cost-effectiveness plane, and a cost-effectiveness acceptability curve will illustrate the probability that the intervention is cost-effective compared to an alternative, given different values of willingness to pay for a unit of health benefit [54].

### Predictors

It is hypothesized that the level of depression treatment resistance (measured by the MSM [23]), childhood adversity (assessed on the CTQ [45, 55]), and expectancies for depressive symptom change (assessed on the pessimistically worded statements in the Depression Change Expectancy Scale [56, 57]) will predict the effect of treatment on depression symptoms in both treatment groups (for description of the assessment tools, please see study protocol [21]. This will be investigated by adding a covariate for MSM to the primary analysis model, as well as an interaction term for Therapy:MSM (see Box 5, Additional file 3). Due to its exploratory nature, this analysis will be based on the per-protocol population, without imputation of missing data.

### Mediators

**Fig. 2:**
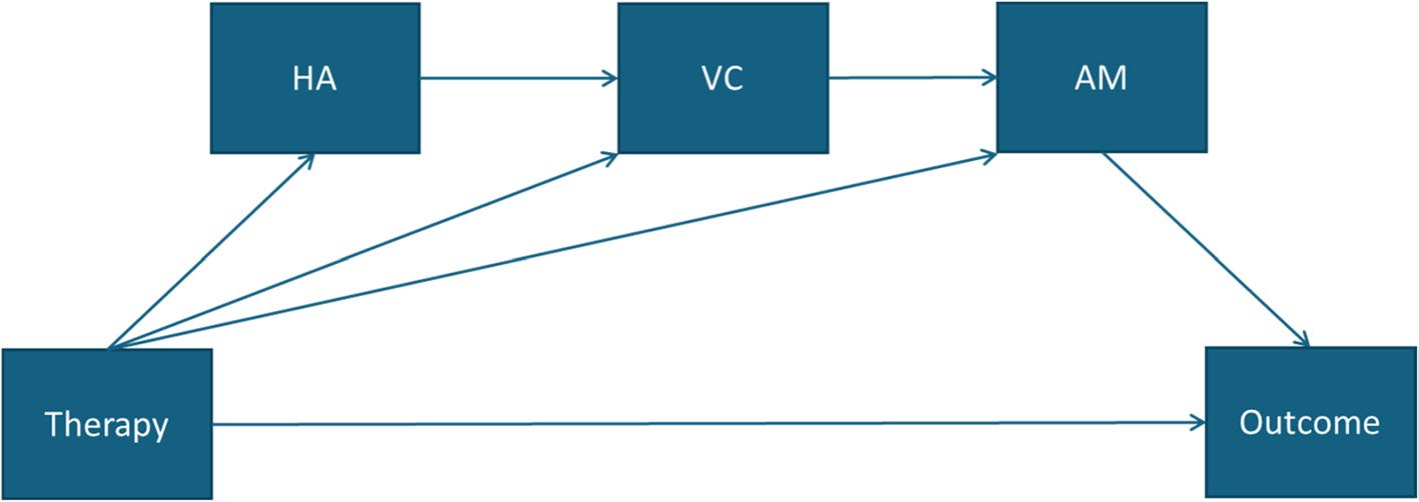
Proposed mediation model HA healthy adult, VC vulnerable child, AM authority modes (demandingand critical authority modes pooled)

ST is theorized to work through changing early maladaptive schemas; however, recent work has focused more on coping with schemas through working with schema modes [16]. We hypothesize that ST will increase the Healthy Adult Mode and minimize the Vulnerable Child Mode and the Demanding/Critical and Punitive authority modes (measured on the SMI; see Fig. 2). An exploratory mediation analysis will be performed to investigate the relative contribution by therapy type of changes in the four mentioned schema modes on depression symptoms as measured by the HAMD-6.

The exposure is, thus, the binary variable, therapy, with levels ST and TAU. The mediators investigated individually are schema modes Vulnerable Child, Punitive authority and Demanding/Critical authority (the two authority modes pooled) for which we expect lower scores under ST as compared to TAU, and Healthy Adult mode for which we expect higher scores under ST as compared to TAU. The outcome is HAMD-6, thus the same as the primary outcome of the main analysis. Due to its exploratory nature, this analysis will be based on the per protocol population, without imputation of missing data. The code is shown in Box 6 (Additional file 3).

### Details of statistical packages to be used to carry out the analyses

For all analyses except for the health economic analyses, R version 4.5.1 will be used [58], with package RefBasedMI for the imputation of continuous missing data [59, 60], and bootImpute [61] and dejaVu [62] for the sensitivity analysis. Lavaan will be used for the mediation analysis [63]. Stata version 18 will be used for the health economic analyses. [64].

## Discussion

The purpose of this paper is to present a detailed, predefined statistical analysis plan in order to minimize bias due to selective selection, analyses, and reporting of outcomes. Important strengths and limitations of the trial and its methods have been presented in the trial protocol [21]. We will now add a few points regarding the strengths and limitations of the analytical and statistical methods.

### Strengths

Methodological strengths for this trial include the following: Having predefined a statistical analysis plan minimizes the potential for data-driven and/or selective outcome reporting. Further, analyses are made according to the intention-to-treat principle, making up for potential bias due to patient characteristics that increase drop-out. We assume normal distribution of the data and specific mechanisms of missingness and dropout. A number of sensitivity analyses have been planned to assess the robustness of the results with regard to these assumptions, including an analysis that treats the primary outcome as an event count.

### Limitations

While power calculations have been made to secure adequate statistical power for primary and secondary outcomes, these calculations were based on treatment effects and their standard deviations from population and interventions that were not exactly identical to those of the current trial [26–28, 35, 65]. This might create inaccuracies in power estimations, potentially skewing the estimation of statistical significance differences between treatment groups. Also, subgroup analyses and exploratory analyses are planned, which are probably underpowered. At the same time, there is a Type 1 error inflation with each additional significance test. This will be taken into account when interpreting the results. Further, while the secondary outcomes are planned to exhibit adequate power, we are not adjusting secondary outcome results for multiple testing, affecting the certainty of findings. This means that conclusions and recommendations based on the results of this trial are made based on the primary outcome only. Finally, given the vulnerable state of the trial population, a substantial amount of missing data can be expected. While this will be handled appropriately as described above, missing data in clinical trials can create validity problems that cannot be corrected for [66]. This will be considered an important potential limitation when reporting the results of our trial.

## Supplementary Information

Additional file 1: Statistical Analysis Plan (SAP) Checklist v 1.0 2019

Additional file 2: Table reporting of baseline characteristics

Additional file 3: Code for analyses to be used in R, version 4.5.1
Additional file 4.

## Data Availability

The datasets used and/or analyzed during the current study are available from the corresponding author on reasonable request.

## Abbreviations

CTQ: Childhood Trauma Questionnaire
DAR: Dimensions of Anger Reactions Scale
DCES-P: Depression Change Expectancy Scale–Pessimistic scale
DSM: Diagnostic and Statistical Manual of Mental Disorders
DSMC: Data Safety Monitoring Committee
DRG: Diagnostic related group
EQ-5D-5L: European Quality of Life 5 Dimensions 5 Level Version
HAMD-6: Hamilton Rating Scale for Depression–6-item version
HA: Healthy adult
ICD-10: International Classification of Diseases, 10th Revision
ICER: Incremental cost-effectiveness ratio
ICH-GCP: International Conference on HarmonisationGood Clinical Practice
INSPIRE-O: Brief INSPIRE for personal recovery (Outcome version)
MAP-9: Metacognitive Anger Processing Scale (9 items)
MCID: Minimal clinically important difference
MDD: Major depressive disorder
M.I.N.I.: Mini International Neuropsychiatric Interview
MI: Multiple imputation
MNAR: Missing not at random
MSM: Maudsley Staging Model
NEQ: Negative Effects Questionnaire
PSYCHLOPS: Psychological Outcome Profiles
PTQ: Perseverative Thinking Questionnaire
QALY: Quality-adjusted life year
RCT: Randomized controlled trial
REDCap: Research Electronic Data Capture
SAP: Statistical analysis plan
SCL-10: Symptom Checklist-10
SD: Standard deviation
SMI 1.1: Schema Mode Inventory version 1.1 (full version)
ST: Schema therapy
TAU: Treatment as usual
VC: Vulnerable child
WHO5: WHO-5 Well-Being Index
WSAS: Work and Social Adjustment Scale
YSQ S.3: Young Schema Questionnaire Short form, version 3
