# Supplementary material for: Statistical analysis plan for a parallel group randomized clinical trial comparing schema therapy versus treatment as usual for outpatients with difficult-to-treat depression (DEPRE-ST)
Source: Trials. 2025 Sep 1;26:334. doi: 10.1186/s13063-025-09012-4 (PMC12403386; doi:10.1186/s13063-025-09012-4)
Supplement: Supplementary file 2 — Additional file 2: Table reporting of baseline characteristics [file 13063_2025_9012_MOESM2_ESM.docx]

# Reporting of baseline characteristics

|  |  | Treatment A (n= xx) | Treatment B (n=x) |
| --- | --- | --- | --- |
| Age (years) | Mean (SD) |  |  |
| Sex | Female |  |  |
|  | Male |  |  |
|  | Other |  |  |
| Country of origin | Danish |  |  |
|  | Non-Danish (Western) |  |  |
|  | Non-Danish (non-Western) |  |  |
| Civil status | Married |  |  |
|  | Living together |  |  |
|  | Single (not married) |  |  |
|  | Single (separated, divorced) |  |  |
|  | Single (widow, widower) |  |  |
|  | Other |  |  |
|  | In a relationship (not living together) |  |  |
| Living situation | Alone |  |  |
|  | With partner |  |  |
|  | With children |  |  |
|  | Other |  |  |
| Number of children (if any) | Mean (SD) |  |  |
| Level of education | No vocational education |  |  |
|  | Other vocational education |  |  |
|  | Higher education (short) |  |  |
|  | Higher education (medium long) |  |  |
|  | Higher education (long) |  |  |
|  | Other |  |  |
| Current job situation | Self-employed |  |  |
|  | Employee (unskilled) |  |  |
|  | Employee (skilled) |  |  |
|  | Full-time employee |  |  |
|  | Student |  |  |
|  | Stay-at-home |  |  |
|  | Parental leave |  |  |
|  | Retired |  |  |
|  | Unemployed |  |  |
|  | Disability pension |  |  |
|  | Sick leave |  |  |
|  | Other |  |  |
| Yearly income of patient | Below 300.000 DKK |  |  |
|  | 300.000-600.000 DKK |  |  |
|  | 600.000-900.000 DKK |  |  |
|  | Above 900.000 DKK |  |  |
| Yearly income of household | Below 300.000 DKK |  |  |
|  | 300.000-600.000 DKK |  |  |
|  | 600.000-900.000 DKK |  |  |
|  | Above 900.000 DKK |  |  |
| Current psychosocial stressors | % |  |  |
| Number of depressions (including the present) | Mean (SD) |  |  |
| Age at the time of the first depression | Mean (SD) |  |  |
| Number of years since first depression | Mean (SD) |  |  |
| Duration of former psychiatric treatment (months) | Mean (SD) |  |  |
| Depression diagnosis from electronic patient journal | F32.1 |  |  |
|  | F32.2 |  |  |
|  | F32.3 |  |  |
|  | F32.8 |  |  |
|  | F32.9 |  |  |
|  | F33.1 |  |  |
|  | F33.2 |  |  |
|  | F33.3 |  |  |
| Other diagnoses from electronic patient journal | Anxiety disorders |  |  |
|  | ADHD |  |  |
|  | PTSD |  |  |
|  | OCD |  |  |
|  | Eating disorders |  |  |
|  | Other |  |  |
| Comorbid diagnoses (M.I.N.I interview) | No comorbid diagnosis |  |  |
|  | Anxiety disorders |  |  |
|  | PTSD |  |  |
|  | OCD |  |  |
|  | Eating disorders |  |  |
|  | Alcohol or drug addiction |  |  |
|  |  |  |  |
| Severity of current depression (HAMD-6) | Moderate depression score 9-11 (Mean, SD) |  |  |
|  | Medium to severe depression without psychotic symptoms score 12-22 (Mean, SD) |  |  |
|  | Total Mean (SD) |  |  |
| Duration of current depression | Acute (less than 12 months) |  |  |
|  | Subacute (13-24 months) |  |  |
|  | Chronic (more than 2 years) |  |  |
| Current and former intake of psychotropic medicine in current depressive episode | No medicine |  |  |
|  | SSRI |  |  |
|  | TCA |  |  |
|  | MAO |  |  |
|  | TeCA |  |  |
|  | SNRI |  |  |
|  | Other antidepressant |  |  |
|  | Other medicine |  |  |
|  | Augmentation |  |  |
| Maudsley Staging Model score | Mean (SD) |  |  |
| Number of different antidepressants (at least 4 weeks) | Mean (SD) |  |  |
| Number of different therapy attempts | Mean (SD) |  |  |
| Expectancy for change in depression (measured with DCES) | Mean (SD) |  |  |
| Exposure to childhood trauma (measured with CTQ) | Mean (SD) |  |  |
| Number of days from randomization to all assessment time points – 6, 12, and 24 months after randomization | 6 months  (Mean SD) |  |  |
|  | 12 months |  |  |
|  | 24 months |  |  |

ADHD = Attention-Deficit/Hyperactivity Disorder; CTQ = Childhood Trauma Questionnaire; DCES = Depression Change Expectancy Scale; DKK = Danish kroner; HAMD-6 = Hamilton Depression Rating Scale – 6 item version; ICD-10 = International Classification of Diseases, 10th Revision; M.I.N.I = Mini International Neuropsychiatric Interview; MAO = Monoamine Oxidase Inhibitor; OCD = Obsessive-Compulsive Disorder; PTSD = Post-Traumatic Stress Disorder; SD = Standard Deviation; SNRI = Serotonin–Norepinephrine Reuptake Inhibitor; SSRI = Selective Serotonin Reuptake Inhibitor; TCA = Tricyclic Antidepressant; TeCA = Tetracyclic Antidepressant.
