# Supplementary material for: Statistical analysis plan for a parallel group randomized clinical trial comparing schema therapy versus treatment as usual for outpatients with difficult-to-treat depression (DEPRE-ST)
Source: Trials. 2025 Sep 1;26:334. doi: 10.1186/s13063-025-09012-4 (PMC12403386; doi:10.1186/s13063-025-09012-4)
Supplement: Supplementary file 3 — Additional file 3: Code for analyses to be used in R, version 4.5.1 Additional file 4. [file 13063_2025_9012_MOESM3_ESM.docx]

*Box 1.* R code for the primary analysis.

library(RefBasedMI)

library(lme4)

library(mice)

library(broom.mixed)

mi <- RefBasedMI(data=d, depvar=CHANGE, treatvar=TREAT, idvar=PATID,

timevar=VISIT, method="J2R", reference=1, M=50, seed=4711)

fit <- with(data=as.mids(mi), lmer(CHANGE ~ T0 + TREAT + (1 | CL),

subset=(VISIT == "12M")))

summary(pool(fit))

*Box 2.* R code for the analysis of available cases.

m <- lmer(CHANGE ~ T0 + TREAT + (1 | CLUSTER), data=d,
 subset=(VISIT == "12M")))

summary(m)

*Box 3.* R code for the sensitivity analysis with negative binomial regression. The code is based on <https://github.com/jwb133/refBasedVar/>

**library**(dejaVu)

**library**(bootImpute)

**library**(MASS)

**library**(RefBasedMI)

impM <- **function**(s, M)

{
 *# Remember Id*

Id0 <- s**$**Id

s**$**Id <- 1**:nrow**(s)

s <- **ImportSim**(

**MakeDejaData**(**subset**(s, select=**c**("Id", "TREAT")), arm="TREAT", Id="Id"),

event.times=**expandEventCount**(count=s**$**observed.events,time=s**$**censored.time),

status="dropout", study.time=365,

censored.time=s**$**censored.time, allow.beyond.study=FALSE)

s**$**data**$**Id <- Id0

*# Fit imputation model*

fit <- **Simfit**(s)

imps <- **Impute**(fit, **weighted_j2r**(trt.weight=0, proper=FALSE), M)

imps_list <- **vector**(mode="list", length=M)

**for**(i **in** 1**:**M)

imps_list[[i]] <- **GetImputedDataSet**(imps, index=i)

imps_list

}

analyseImp <- **function**(s)

{

d1 <- **cbind**(s**$**data, T0=d[**as.character**(s**$**data**$**Id), "T0"])

m <- **glm.nb**(observed.events **~** TREAT **+** T0, data=d1)

**coef**(m)

}

d <- d[d**$**VISIT **==** "12M", ]

d**$**Id <- d**$**PATID

**rownames**(d) = d**$**Id

d**$**observed.events <- d**$**HAMD6

d**$**censored.time = **ifelse**(**is.na**(d**$**observed.events), 1, 365)

d**$**observed.events[**is.na**(d**$**observed.events)] = 0

d**$**T0 <- **as.numeric**(d**$** T0)

imp <- **bootImpute**(d, impM, nBoot=500, nImp=2, M=2)

**bootImputeAnalyse**(imp, analyseImp)

*Box 4.* R code for subgroup analyses.

# Calculate predictor for interaction Gender x Therapy (Rest is unchanged)
d$GxT = factor(ifelse(d$SEX == 1 & d$TREAT == 2, yes="MxDRUG", no="OTHER"))

...

fit <- with(data=as.mids(mi), lmer(CHANGE ~ T0 + SEX + TREAT + GxT + (1 | CL),

subset=(VISIT == "12M")))

...

*Box 5.* R code for the analysis of predictors.

# Does MSM improve prediction in general?
m0 <- lmer(HAMD6 ~ T0 + TREAT + (1 | CL), ...)

m1 <- lmer(HAMD6 ~ T0 + TREAT + MSM + (1 | CL), ...)

# Does MSM improve prediction in specific treatment arms?

m2 <- lmer(HAMD6 ~ T0 + TREAT + TREAT:MSM + (1 | CL), ...)

anova(m0, m1, m2)

*Box 6.* R code for the mediation analysis.

library(lavaan)

d12 <- d[d$VISIT == "12M", ]

model <- '

AM ~ u1*T0 + a1*TREAT + b1*VC

VC ~ u2*T0 + a2*TREAT + b2*HA

HA ~ u3*T0 + a3*TREAT

Outcome ~ u1*T0 + d*TREAT + c*AM

direct := d

indirect := a1*c + a2*b1*c + a3*b2*b1*c

total := direct + indirect'

sem(model, data=d12, cluster="CL")
